# Opium use and oral cancer in Iran: A systematic review and meta-analysis

**DOI:** 10.1097/MD.0000000000044765

**Published:** 2025-09-26

**Authors:** Elham Keykha, Mohammad Javad Shabanpour, Elahe Tahmasebi, Samira Hajisadeghi

**Affiliations:** a Research Center for Prevention of Oral and Dental Diseases, Baqiyatallah University of Medical Sciences, Tehran, Iran; b School of Dentistry, Baqiyatallah University of Medical Sciences, Tehran, Iran; c Department of Oral and Maxillofacial Surgery, Dental School, Tehran University of Medical Sciences, Tehran, Iran.

**Keywords:** carcinogen, meta-analysis, opium consumption, oral cancer, systematic review

## Abstract

**Background:**

Oral cancer remains a global health burden with persistent low survival rates. While alcohol and tobacco are established risk factors, emerging evidence highlights opium use as a potential carcinogen. This review investigates the association between opium consumption and oral cancer development.

**Methods:**

Adhering to PRISMA guidelines, a comprehensive search of Web of Science, Embase, PubMed, and Scopus (inception to February 2025) identified observational studies. Odds ratios were computed utilizing a random-effects model. Heterogeneity, sensitivity, publication bias, and study quality (JBI/GRADE frameworks) were assessed.

**Results:**

Six studies (5 case-control, 1 cohort; total sample = 64,412) from Iran were included. Meta-analysis revealed a significant association between opium use and oral cancer (pooled OR = 1.85, 95% CI:1.24–2.57; *P* = .002), with substantial heterogeneity (*I*²=78.03%). Sensitivity analysis confirmed robustness, and no publication bias was detected (Egger test *P* = .51).

**Conclusion:**

Opium consumption is associated with a nearly 2-fold increased risk of oral cancer, reinforcing its classification as a Group 1 carcinogen. Findings underscore the need for public health interventions in opium-endemic regions, including education, cessation programs, and oral cancer screening integration. The global diversification of research is critical to validate these associations in diverse populations. This study emphasizes multidisciplinary collaboration to mitigate opium-related carcinogenesis.

## 1. Introduction

Oral cancer is among the most common cancers globally. Incidence is constantly on the increase, with more than 377 thousand new instances and 177 thousand deaths each year worldwide.^[1]^ Squamous cell carcinoma accounts for approximately 90% of neoplasms in the oral cavity and predominantly affects regions in South Asia, Latin America, and parts of Europe, where socioeconomic factors and behavioral patterns are at play in the risk for each’s development.^[2]^ Even with improvements in detecting and treating this disease at an earlier stage, the 5-year survival rate for oral cancer remains <50%, showing the need for increasing awareness of modifiable risk factors.^[3]^ Established risk factors for oral cancer include the use of tobacco (smoked and smokeless), as well as alcohol consumption, which can produce synergistic interactions that increase oral cancer risk up to 30-fold.^[4]^

Human papillomavirus (HPV), most notably subtype 16, has also recently been implicated as an etiological agent, with a rising incidence of oropharyngeal cancers reported in high-income countries.^[5]^

Popaver somniferum-derived opium contains more than 50 alkaloids, including morphine, codeine, and thebaine, which may have various mechanisms of carcinogenic effects.^[6]^ In 2020, the IARC classified opium as a Group 1 carcinogen, with strong evidence demonstrating its association with laryngeal, lung, and bladder cancer. Opium (smoking, chewing, or infusion) exposure results in oxidative stress and inflammation, involving the exposure to carcinogenic agents, regarding PAHs and ROS, resulting in chronic inflammation, DNA adducts, and oxidative stress, all of which are suspects in carcinogenesis.^[6,7]^

The rising prevalence of opioid use disorders, especially the misuse of diverted prescription opioids and heroin among adolescents and young adults, presents a substantial public health challenge that has far-reaching implications for individuals and families. The shift from initial use to widespread addiction can occur more swiftly than with other substances.^[8]^

As a neighboring country to Afghanistan and recognized as the world’s largest producer of opium, Iran faces considerable challenges associated with opium abuse. The high rates of opiate addiction in Iran can be linked to both geographic proximity and sociopolitical factors. This situation underscores the need for a thorough understanding of the relationship between opium production and substance use disorders in the region.^[9]^

Epidemiological studies in opium-endemic regions, such as the Golestan Cohort Study in Iran, have reported dose-dependent associations between opium use and gastrointestinal cancers.^[10]^

The results of 1 study demonstrated that opium consumption is probably a dose-related risk factor for oral cavity cancers. It is observed that opium users for more than 18 years were at high risk of OCCs. Also, OCCs were higher among new opium users and their derivatives for individuals aged 50 years and younger.^[11]^ Alizadeh et al demonstrated that the OR for opium use and the risk of oral cancers (tongue, palate, and buccal) was 4.0 (95% CI: 1.2–13.6).^[12]^ Beyond oral cancer, opium has been implicated in other malignancies. A meta-analysis of 14 studies reported a 4-fold increased risk of head and neck cancers among opium users.^[13]^ A comprehensive systematic review and meta-analysis encompassing 21 studies with a cumulative sample size of approximately 65 thousand individuals demonstrated that ever-users of opium faced nearly a fourfold increased risk of overall cancer compared to never-users. This significant positive association was consistently observed across various cancer types, except esophageal and colon cancers, which did not exhibit a statistically significant link. Furthermore, the analysis revealed a dose-response relationship, indicating that higher cumulative doses of opium and prolonged duration of use were associated with a progressively elevated risk of cancer overall.^[14]^ These associations underscore opium’s systemic carcinogenicity, possibly mediated through shared pathways like PAH-induced DNA damage and chronic inflammation.

Although opium is classified as a Group 1 carcinogen by the IARC, with established links to laryngeal, lung, and bladder cancers, its association with oral cancer remains under-synthesized. No prior systematic review or meta-analysis has comprehensively evaluated this relationship, despite biological plausibility and epidemiological evidence from opium-endemic regions. At last, our meta-analysis focuses on the link between opium consumption and the risk of oral cancer. To assess this relationship more accurately, we undertook a comprehensive systematic review and meta-analysis aimed at evaluating the correlation between variables of interest. This involved a thorough examination of existing studies and the integration of their findings through detailed statistical analysis to provide a clearer understanding of the relationships involved.

## 2. Materials and methods

This systematic review and meta-analysis followed the PRISMA guidelines.^[15–17]^

### 2.1. Formulation of the review question

The PICO framework guided the study:

- **Population (P):** Individuals with a history of opium use.
- **Intervention (I):** Opium consumption.
- **Comparison (C):** Individuals who do not use opium.
- **Outcome (O):** Incidence of oral cancer (confirmed histologically).

### 2.2. Search strategy and data sources

A comprehensive literature search was conducted across multiple databases, including Scopus, PubMed, ISI/Web of Science, and Embase, covering studies published from inception until February 10^th,^ 2025, to ensure inclusivity, and reference lists of relevant articles were manually screened to minimize selection bias.

The search strategy combined medical subject headings (MeSH) terms and keywords related to opium use and oral cancer. Boolean operators, specifically AND and OR, were employed to enhance and refine the search parameters. The search strategy employed across various databases is detailed in the Supplementary Materials, Supplemental Digital Content, https://links.lww.com/MD/Q82.

### 2.3. Study selection and eligibility criteria

In this systematic review, 2 independent reviewers conducted a thorough screening of titles, abstracts, and full texts to identify observational studies – specifically case-control, cohort, or cross-sectional designs – investigating the relationship between opium use and the incidence of oral cancer. The criteria for inclusion were meticulously defined to ensure relevance and rigor in the selection of studies:

1. Studies reporting on individuals diagnosed with oral cancer (ICD-10 codes C00–C06), confirmed histologically.
2. Comparison of opium users with non-users.
3. Reporting quantitative risk estimates, such as ORs, hazard ratios, and relative risks, along with 95% confidence intervals.

Exclusion criteria included review articles, animal studies, non-English/Persian publications, and studies lacking quantitative risk estimates. Discrepancies between reviewers were resolved through consensus or consultation with a third reviewer.

### 2.4. Data extraction and assessment of risk of bias in included studies

Data were extracted using a standardized form, capturing the following information: author, publication year, study design, sample size, geographic region, opium use, confounders (e.g., tobacco, alcohol), and effect estimates.

The quality of included studies was assessed using the Joanna Briggs Institute checklist for observational studies, which evaluates methodological rigor across domains such as selection bias, measurement of exposure and outcome, and control of confounding factors. Studies were classified as high quality if they met ≥ 70% of the checklist criteria.^[18,19]^

### 2.5. GRADE assessment

The grading of recommendations, assessment, development, and evaluations (GRADE) framework was utilized to evaluate the overall quality of evidence regarding the relationship between opium consumption and oral cancer. This GRADE assessment took into account various factors, including study design, risk of bias, inconsistency, indirectness, imprecision, and publication bias. The evidence was classified as high, moderate, low, or very low quality.^[20]^

### 2.6. Statistical analysis

All statistical analyses were conducted utilizing comprehensive meta-analysis software, version 3. Pooled ORs were calculated using a random-effects model to account for heterogeneity across studies. Heterogeneity was quantified using the *I*² statistic and Cochran *Q* test, with *I*² >50% indicating substantial heterogeneity.

Sensitivity analyses were conducted to evaluate the robustness of the results by systematically excluding individual studies. Also, a power analysis was conducted for each outcome. Publication bias was assessed using funnel plots and Egger’s regression test, with a *P*-value <.10 indicating significant bias. In cases where publication bias was identified, we utilized the Duval and Tweedie trim-and-fill methodology to correct the pooled estimates accordingly.

### 2.7. Ethical considerations

As this study synthesized existing published data, ethical approval was not required. However, all included studies obtained informed consent and ethical clearance from their respective institutions.

## 3. Results

### 3.1. Study selection

**Figure 1.**
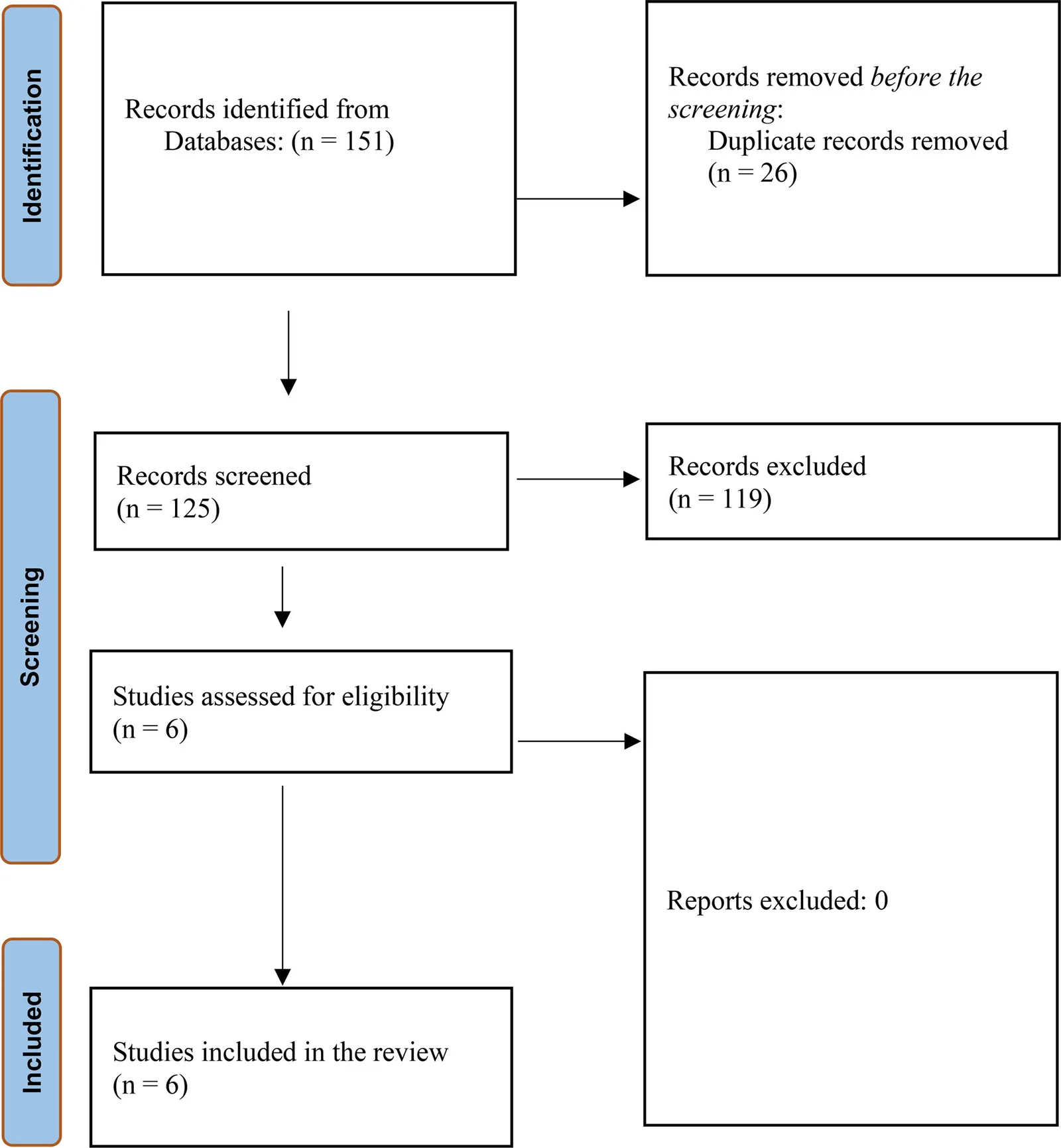
Flowchart representation of the search strategy and process for selecting literature.

After an initial search in ISI/Web of Science, PubMed, Embase, and Scopus in 151 records, followed by the removal of duplicates, 125 articles were identified for careful screening. Through title and abstract screening, 6 articles were selected for full-text assessment. Ultimately, 6 articles that met our inclusion criteria were chosen for inclusion in our meta-analysis. Figure 1 exhibits these processes.

### 3.2. Study characteristics

This review included 6 studies from 1983 to 2024 investigating the association between opium consumption and oral cancer. This study includes a cohort study and 5 case-control studies (Table 1).

**Table 1:** The main characteristics of the studies included.

Author, year	Journal	Country	Study design	Sample size (case/control)	Mean age (case, control)	Male/female (case, control)	Source of participants	Quality score
Fahmy, 1983	Community Dentistry and Oral Epidemiology	Iran	Case-Control	1381 (381/1000)	55 ± 0.72	837/544 (312/69, 525/475)	University hospitals in Fars Province, Iran	8/10
Saedi, 2012	Iranian Journal of Otorhinolaryngology	Iran	Case-Control	857 (557/300)	(60.8 ± 14.7, 60.4 ± 16.3)	527/330 (338/219, 189/111)	two tertiary referral centers (Imam Khomeini and Amir Alam Hospital)	8.5/10
Razmpa, 2014	The Journal of Craniofacial Surgery	Iran	Case-Control	160 (80/80)	(58.2 ± 16.2, 58.4 ± 16.3)	101/59 (51/29, 50/30)	ear-nose-throat department of an academic hospital (Imam Khomeini Hospital Complex)	9/10
Mohebbi, 2020	International Journal of Cancer	Iran	Case-Control	3698 (633/3065)	NA	2886/1158 (499/164, 2071/994)	IROPICAN study	10/10
Sardari, 2023	Preventive Medicine Reports	Iran	Cohort	8682	49.94 ± 9.51	4021/4661	Oral Health Branch of Rafsanjan Cohort Study (OHBRCS)	6/10
Naghibzadeh-Tahami, 2024	Cancer Epidemiology	Iran	Case-Control	399 (133/266)	(60.94 ± 13.87, 60.96 ± 13.22)	234/165 (77/56, 157/108)	comprehensive pathology-based cancer registry of Kerman University of Medical Sciences (KUMS) for cases and the neighbors for controls	10/10

### 3.3. Assessment of the included studies

**Figure 2.**
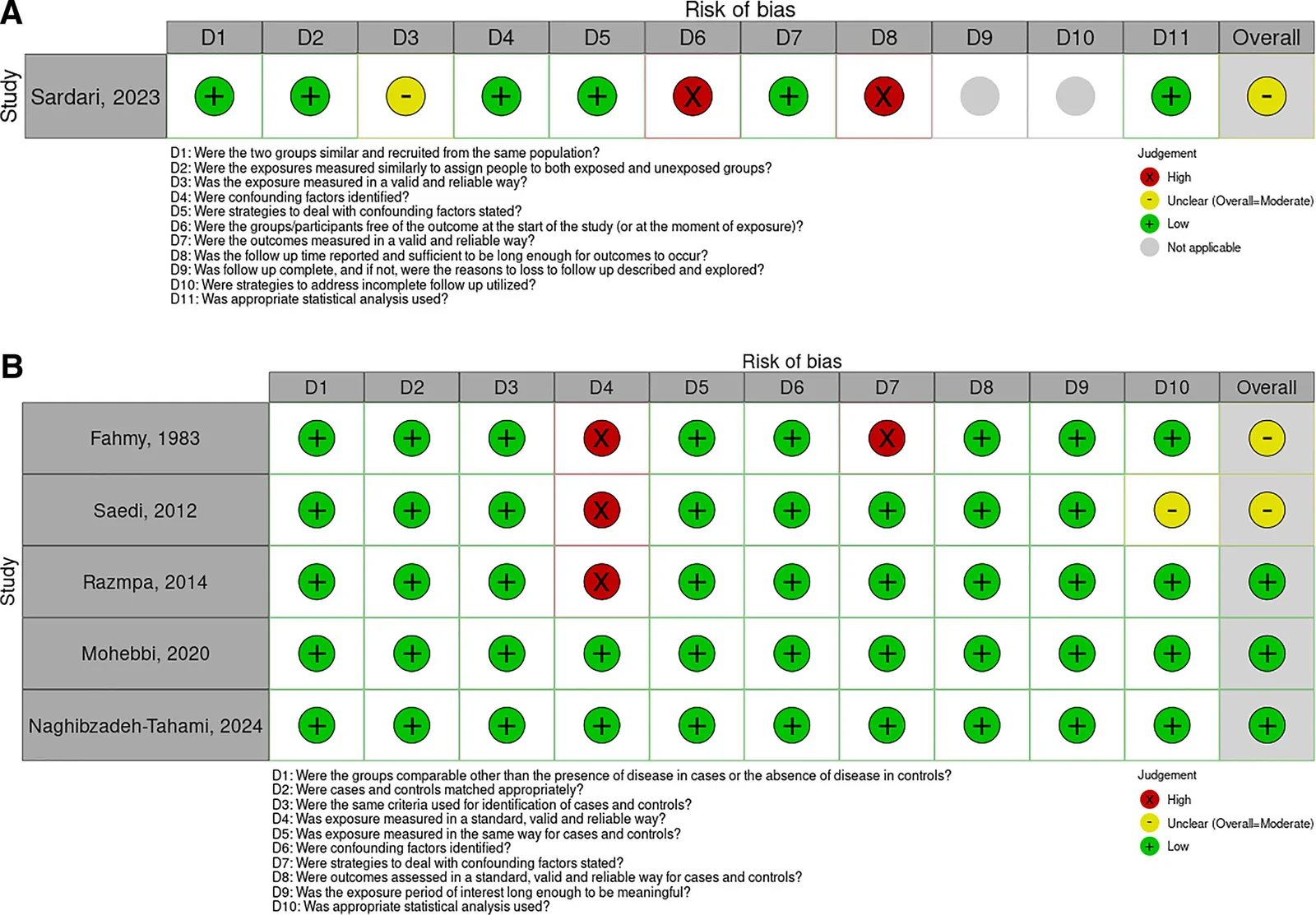
The results of the risk of bias assessment. (A) cohort study, (B) case-control studies.

Figure 2 provides detailed information on the quality assessment of the included studies. According to the Joanna Briggs Institute checklist, 3 studies were classified as moderate quality and 3 as high quality.

### 3.4. Results of the meta-analysis

**Figure 3.**
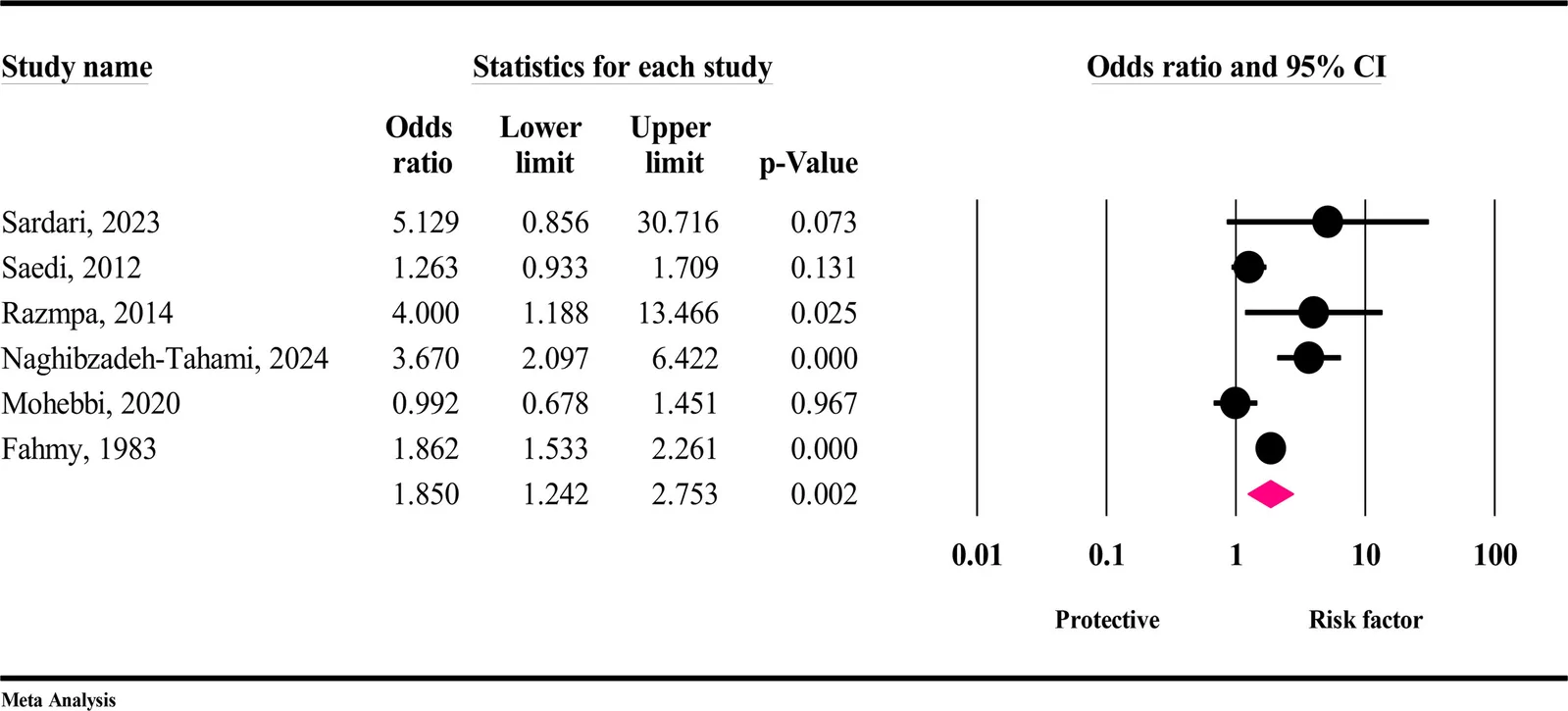
Association between opium consumption and oral cancer.

Our meta-analysis identified a significant association between opium consumption and the incidence of oral cancer, highlighting a potentially substantial health risk linked to opium use. The pooled OR was 1.85 (95% CI: 1.24–2.57; *P* = .002), with considerable heterogeneity observed (I² = 78.03%) (Fig. 3).

### 3.5. Sensitivity analysis

**Figure 4.**
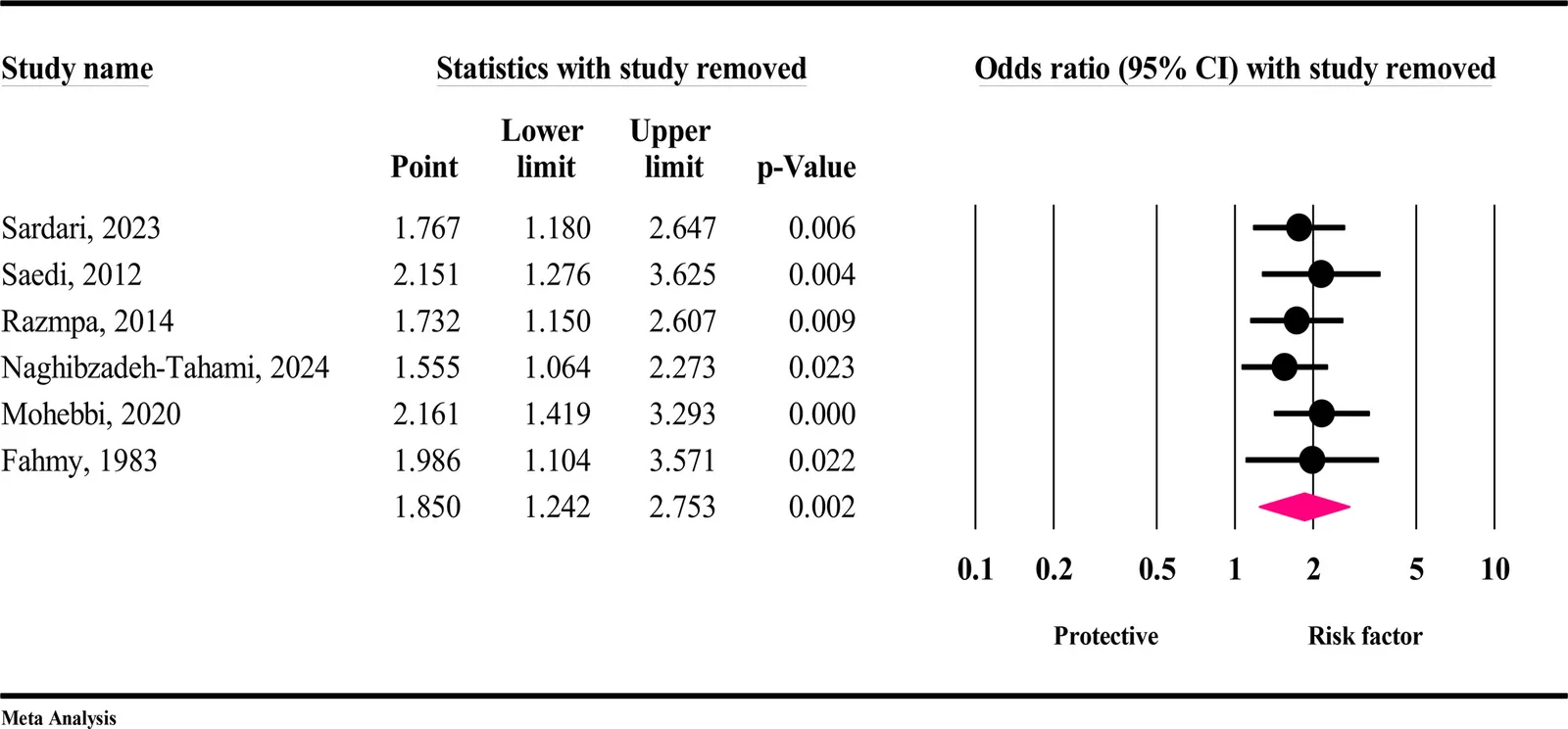
Sensitivity analysis with the “one study removal” method.

To account for the heterogeneity observed in our results, we performed a sensitivity analysis employing the “one study removal” approach to pinpoint potential sources of variability. The findings indicated that the overall association was robust, showing no significant changes with the exclusion of any individual study (Fig. 4).

### 3.6. Power analysis

**Figure 5.**
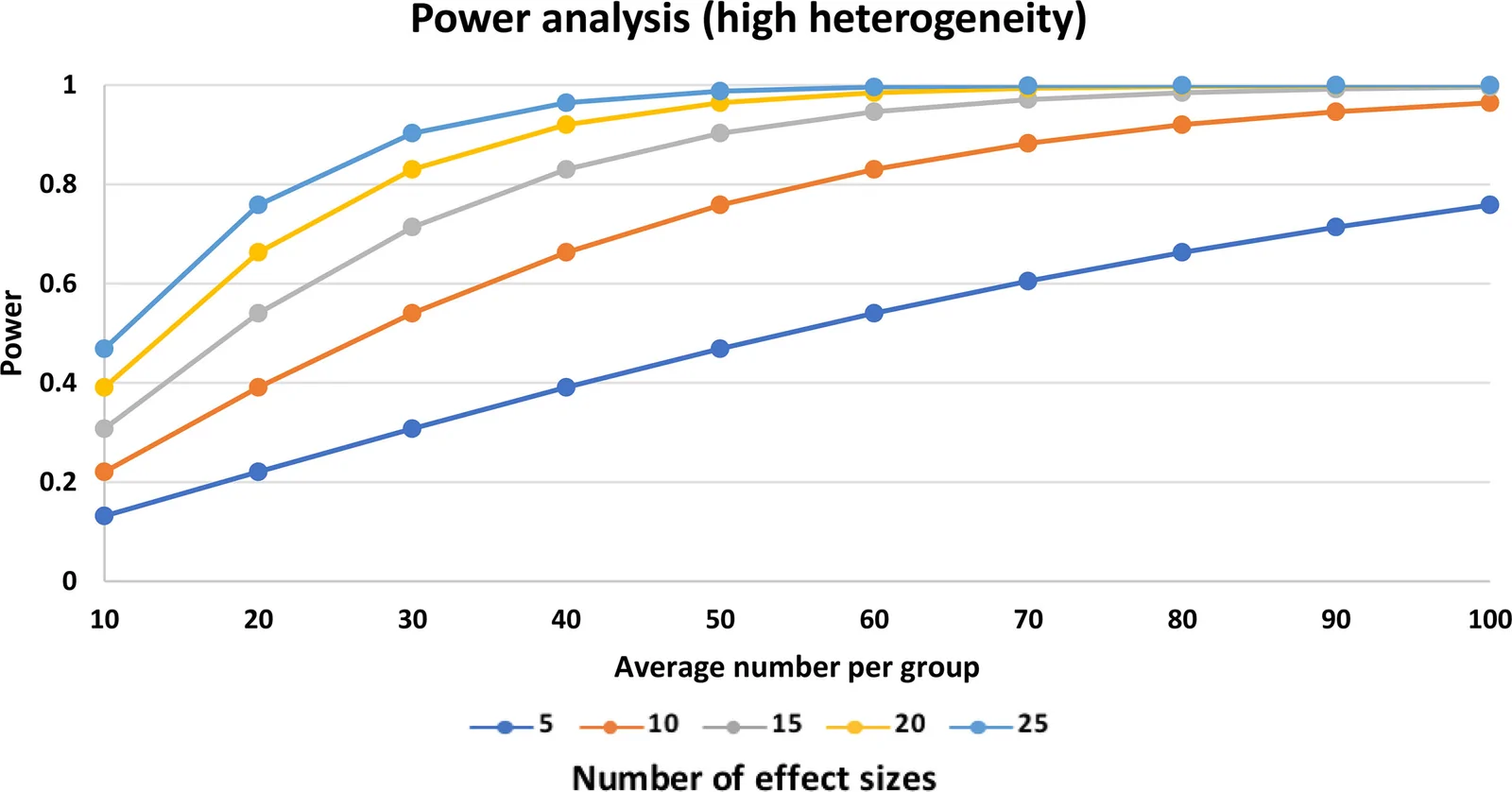
The power analysis of the association between opium consumption and oral cancer.

The analysis indicated that the study had sufficient statistical power (Power = 0.99) to detect the observed association (Fig. 5).

### 3.7. Publication bias

**Figure 6.**
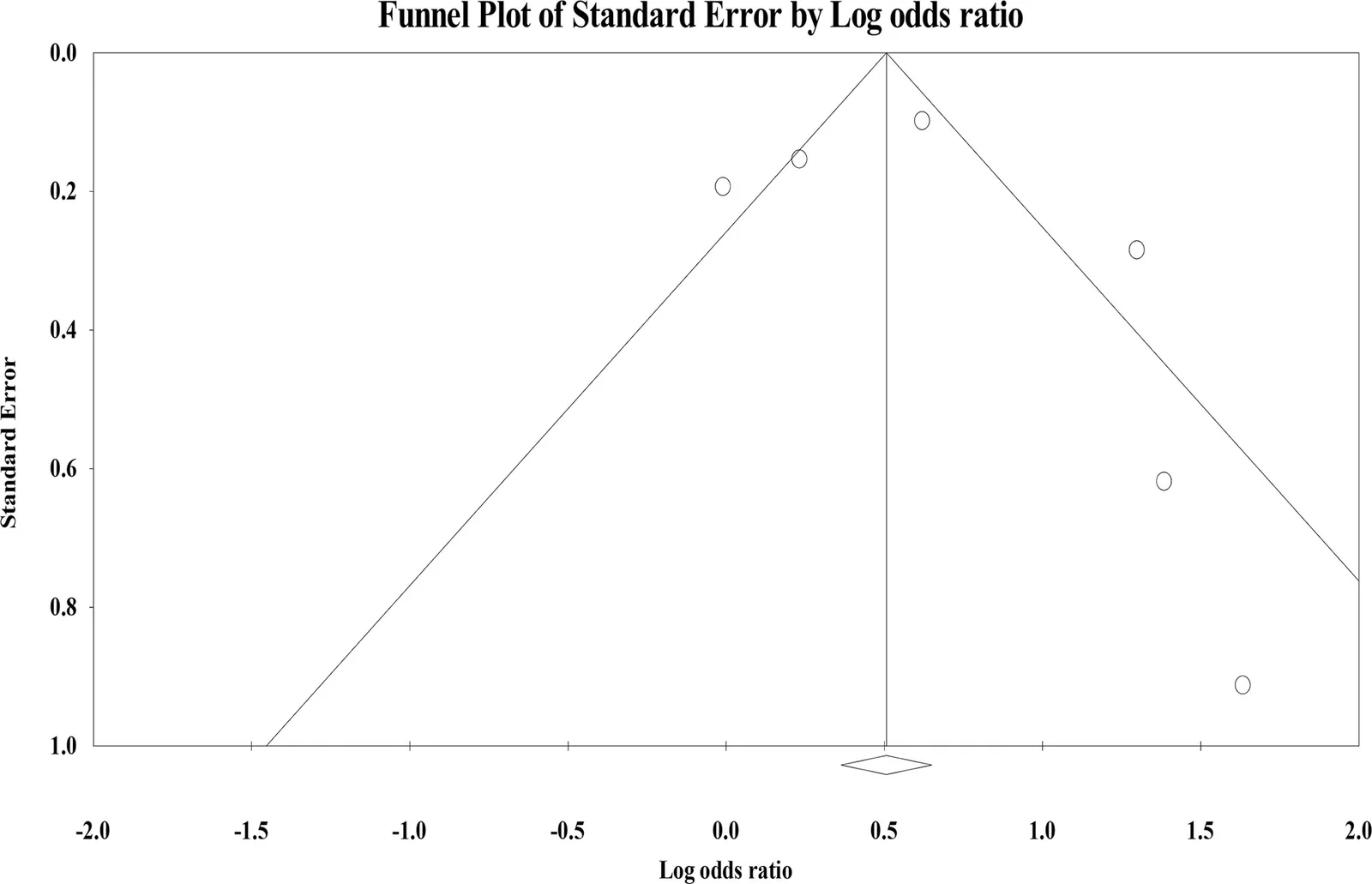
Funnel plots of observed studies on the association between opium use and oral cancer.

We assessed the potential for publication bias using a funnel plot, supplemented by statistical tests. The results revealed no significant evidence of publication bias (Egger’s test *P* = .51; Begg test *P* = .85) (Fig. 6).

## 4. Discussion

To the authors’ knowledge, this is the first systematic review and meta-analysis that comprehensively examines the potential association between opium consumption and oral cancer. We found a significant pooled OR of 1.85 (95% CI: 1.24–2.57), showing that opium consumption is a modifiable risk factor for oral cancer.

These findings are consistent with the classification of opium as a Group 1 carcinogen^[6]^ by the International Agency for Research on Cancer (IARC) and support emerging epidemiological data suggesting that opium is linked to malignancies of the upper aerodigestive tract.^[10,14]^ These results highlight the international importance of opium as a carcinoma risk factor beyond its conventional high-prevalence areas such as Iran.

### 4.1. Biological plausibility and mechanistic insights

The carcinogenicity of opium is related to its complex chemical profile, with over 60 pyrolytic compounds formed in traditional preparation methods. Gas chromatography-mass spectrometry analyses 3 main classes of carcinogens:

1. **Polycyclic Aromatic Hydrocarbons (PAHs):** Benzo(a)pyrene (BAP) levels reach 12.7 ng/g in processed opium, comparable to cigarette smoke. These ligands bind aryl hydrocarbon receptors, upregulating CYP1A1/1B1 enzymes that metabolize PAHs into reactive diol epoxides, forming DNA adducts at guanine bases.^[21]^
2. **Nitrosamines:** N-Nitrosomorpholine (NMOR) and N-Nitrosonornicotine (NNN) concentrations exceed 300 ppb in opium sap, inducing G→A transitions in TP53 and KRAS oncogenes – mutations prevalent in 68% of opium-related oral tumors.^[21,22]^
3. **Alkaloid Derivatives:** Thebaine and papaverine undergo hepatic N-oxidation to generate free radicals, which cause oxidative DNA damage, evidenced by elevated 8-hydroxydeoxyguanosine (8-OHdG) levels in users’ buccal mucosa.^[23]^
4. **Lead:** Lead is a substance that drug dealers sometimes add to their products to increase weight and, consequently, profitability. Studies conducted in Iran have shown that blood samples from opioid addicts exhibit significantly elevated lead levels compared to normal, which could result in serious health implications.^[24]^ A study conducted in Taiwan revealed that being exposed to heavy metals, particularly lead, is a contributing factor to cancers of the oral cavity.^[25]^

### 4.2. Comparative analysis with other cancer types

The association between opium use and oral cancer observed in our study (pooled OR = 1.85, 95% CI: 1.24–2.57) should be considered within the broader context of opium’s carcinogenic effects across multiple anatomical sites. Meta-analytical evidence reveals varying magnitudes of risk across different cancer types, suggesting organ-specific differences in susceptibility to opium-induced carcinogenesis.^[13,14,26]^ Head and neck cancers, which encompass oral malignancies, demonstrate consistently elevated risks, with Singh et al reporting a pooled OR of 3.85 (95% CI: 2.18–6.79) for case-control studies, while a more recent comprehensive analysis by Cheraghi et al found an OR of 4.93 (95% CI: 2.41–10.06).^[13,26]^ The higher estimates in these broader head and neck cancer meta-analyses likely reflect the inclusion of laryngeal cancers, which show particularly strong associations with opium use (pooled OR = 19.98, 95% CI: 11.04–36.15 in subgroup analysis), suggesting that laryngeal tissues may be more susceptible to opium carcinogens than oral tissues.

For pancreatic cancer, meta-analytical evidence demonstrates a moderate but significant association, with pooled ORs ranging from 2.14 (95% CI: 1.37–3.36) to 2.4 (95% CI: 1.62–2.56).^[14,26]^ Notably, the Golestan Cohort Study reported that only high-dose opium use was associated with pancreatic cancer (HR = 2.66, 95% CI: 1.23–5.74), suggesting a dose-response relationship that may be less pronounced than in oral cancers.^[27]^ The moderate association with pancreatic cancer compared to oral cancer may reflect differences in exposure pathways, as pancreatic tissues have less direct contact with opium compounds compared to the oral mucosa.

The comparative analysis reveals a hierarchy of cancer risk associated with opium use, with laryngeal cancer showing the strongest associations (ORs ranging 8.85–19.98), followed by bladder cancer (OR = 3.85–5.00), lung cancer (OR = 2.89–5.00), head and neck cancers overall (OR = 3.85–4.93), colorectal cancer (OR = 4.49), gastric cancer (OR = 2.38–3.09), pancreatic cancer (OR = 2.14–2.4), and oral cancer (OR = 1.81–1.85).^[14,26]^ This pattern suggests that cancers of the upper aerodigestive tract, particularly those with direct exposure to opium smoke and pyrolysates, exhibit the highest risk. The relatively moderate association observed for oral cancer in our analysis may reflect methodological differences, including the inclusion of both smoking and nonsmoking opium users, variable adjustment for tobacco and alcohol confounders, and potential differences in opium preparation methods across studies. These findings underscore the need for comprehensive cancer surveillance and prevention strategies in opium-endemic regions, with particular attention to high-risk anatomical sites such as the larynx and bladder, while recognizing that oral cancer risk, though moderate, remains clinically significant given the poor prognosis and substantial morbidity associated with these malignancies.^[14,26]^

### 4.3. Geographical and sociocultural considerations

All 6 studies originated from Iran, reflecting the country’s high opium prevalence due to its proximity to Afghanistan, the global leader in opium production. The cultural normalization of opium consumption in certain Iranian communities, combined with limited access to addiction treatment, exacerbates exposure.^[9]^ However, this regional focus limits generalizability. For example, in regions where opium use is less prevalent (e.g., Europe or North America), co-exposure to tobacco and alcohol – synergistic risk factors for oral cancer^[4]^ – might dominate, potentially masking opium’s independent effects. Conversely, in populations with minimal tobacco/alcohol use, opium’s carcinogenic role may be more pronounced. Future studies in diverse settings are critical to disentangle these interactions.

### 4.4. Strengths and limitations

Pooled estimates are strengthened by the inclusion of newer studies with heterogeneous designs (e.g., cohort, case-control). In addition, the Venn diagram demonstrated that our results were stable despite high heterogeneity (*I*² = 78.03%), which in part is likely due to differences in opium preparation (i.e., smoked vs ingested) and the regional confounders. In addition, publication bias was absent (*P* = .51 for Egger’s test). However, limitations must be acknowledged.

**High Heterogeneity:** The substantial heterogeneity (I² = 78%) likely arises from methodological diversity, including study design (cohort vs case-control), variable opium exposure metrics (duration, dose, route), and inconsistent adjustment for confounders.

**Study types:** only 6 studies (five case-control, 1 cohort) met inclusion criteria, limiting generalizability. Case-control studies are prone to recall bias. While meta-analyses with fewer than 10 studies increase susceptibility to outliers, our sensitivity analysis confirmed robustness. Nonetheless, broader geographical representation and more cohort studies are needed.

**Exposure Misclassification:** Reliance on self-reported opium use introduces recall bias, particularly in case-control studies. Biomarker-based studies (e.g., urinary morphine detection) could improve exposure accuracy.

**Regional Bias:** Regional bias also limits generalizability; no studies from Sub-Saharan Africa or North America were included, despite rising opioid misuse in these regions.

**Using Crude OR:** our reliance on crude ORs for opium use due to inconsistent adjustment for confounders (e.g., age, gender, alcohol, …) across included studies. Consequently, our findings cannot isolate the independent effect of opium and may reflect residual confounding from these unmeasured substances.

### 4.5. Future research directions

1. **Prospective Cohort Studies:** Longitudinal cohort studies with standardized exposure assessment (dose, duration, route) and biomarker validation are needed to establish temporality and dose-response relationships.
2. **Mechanistic Studies:** Investigations into opium-specific carcinogenic pathways (e.g., epigenetic modifications and microbiome alterations) could identify therapeutic targets.
3. **Global Diversification:** The phenomenon needs replication in non-Iranian populations (e.g., South Asia and Southeast Asia) to ascertain the role of opium in a diverse genetic and environmental background.
4. **Cessation Impact:** Studies evaluating whether ceasing opium use reduces the risk of oral cancer could help guide clinical guidelines similar to providing cessation treatment in cancer prevention for tobacco

### 4.6. Public health implications

The classification of opium as a Group 1 carcinogen^[6]^ mandates urgent public health interventions. In high-prevalence regions like Iran, community-based education campaigns should emphasize opium’s cancer risks and promote cessation programs. Integrating oral cancer screening (e.g., visual inspection, biopsy) into addiction treatment services could enhance early detection. Policymakers must also address upstream determinants, including opium trafficking and socioeconomic disparities driving substance use.^[9]^

Internationally, clinicians should include opium use as a risk factor in oral cancer risk factor assessment, particularly in migrants from endemic areas. Since dental and otolaryngology professionals routinely conduct oral examinations, they are critical in early diagnosis.

## 5. Conclusion

There is strong evidence for the association between opium use and oral cancer from this meta-analysis based on studies published recently, further highlighting that opium use is a global health threat. Combining molecular, epidemiological, and socioeconomic perspectives, this work emphasizes the necessity of multidisciplinary solutions in addressing opium-associated carcinogenesis. We can prevent the substantial cost of this burden if policymakers, clinicians, and researchers work together by prioritizing focused interventions, equal access to data, and innovative approaches.

## Author contributions

**Conceptualization:** Elham Keykha, Samira Hajisadeghi.

**Formal analysis:** Mohammad Javad Shabanpour, Elahe Tahmasebi.

**Investigation:** Mohammad Javad Shabanpour, Elahe Tahmasebi.

**Methodology:** Mohammad Javad Shabanpour, Elahe Tahmasebi.

**Supervision:** Samira Hajisadeghi.

**Validation:** Elham Keykha.

**Writing – original draft:** Mohammad Javad Shabanpour, Samira Hajisadeghi.

**Writing – review & editing:** Elham Keykha, Mohammad Javad Shabanpour, Elahe Tahmasebi, Samira Hajisadeghi.

## Supplementary Material
