# Supplementary material for: Opium use and oral cancer in Iran: A systematic review and meta-analysis
Source: Medicine (Baltimore). 2025 Sep 26;104(39):e44765. doi: 10.1097/MD.0000000000044765 (PMC13593065; doi:10.1097/MD.0000000000044765)
Supplement: Supplementary file 1 [file medi-104-e44765-s001.docx]

**February 10^th^ 2025**

**PubMed=26**

("Opium"[Mesh] OR "Opiate Alkaloids"[Mesh]) AND ("Mouth Neoplasms"[Mesh])

**Scopus=100**

TITLE-ABS-KEY ( "Mouth Neoplasm*" OR "Neoplasm*, Mouth" OR "Neoplasm*, Oral" OR "Oral Neoplasm*" OR "Cancer of Mouth" OR "Mouth Cancer*" OR "Cancer*, Mouth" OR "Oral Cancer*" OR "Cancer*, Oral" OR "Cancer of the Mouth" ) AND TITLE-ABS-KEY ( "Opium" OR "Omnopon" OR "Pantopon" OR "Papaveretum" OR "Alkaloid*, Opiate*" OR "Opiate*" OR "Opiate*, Alkaloid*" OR "Alkaloid Opiate*" )

**Web of Science=14**

TS=("Mouth Neoplasm*" OR "Neoplasm*, Mouth" OR "Neoplasm*, Oral" OR "Oral Neoplasm*" OR "Cancer of Mouth" OR "Mouth Cancer*" OR "Cancer*, Mouth" OR "Oral Cancer*" OR "Cancer*, Oral" OR "Cancer of the Mouth" OR "Oral cavity Cancer*") AND TS=("Opium" OR "omnipod" OR "pantodon" OR "Papaveretum" OR "Alkaloid*, Opiate*" OR "Opiate*" OR "Opiate*, Alkaloid*" OR "Alkaloid Opiate*")

**Embase=11**

('mouth neoplasm*':ab,ti OR 'neoplasm*, mouth':ab,ti OR 'neoplasm*, oral':ab,ti OR 'oral neoplasm*':ab,ti OR 'cancer of mouth':ab,ti OR 'mouth cancer*':ab,ti OR 'cancer*, mouth':ab,ti OR 'oral cancer*':ab,ti OR 'cancer*, oral':ab,ti OR 'cancer of the mouth':ab,ti OR 'oral cavity cancer*':ab,ti) AND ('opium':ab,ti OR 'omnopon':ab,ti OR 'pantopon':ab,ti OR 'papaveretum':ab,ti OR 'alkaloid*, opiate*':ab,ti OR 'opiate*':ab,ti OR 'opiate*, alkaloid*':ab,ti OR 'alkaloid opiate*':ab,ti)
